# High-Throughput Phenotyping: Status and Applications in Rice Breeding

**DOI:** 10.3390/plants15131944

**Published:** 2026-06-24

**Authors:** Leonilo Gramaje, Parthiban Thathapalli Prakash, Nia Manlulu, Rogemae Ravela, Monique Corpuz, Alvin Palanog, Norvie Manigbas, Pompe Sta Cruz, Suresh Babu Kadaru, Jose Hernandez

**Affiliations:** 1 Philippine Rice Research Institute, Science City of Muñoz 3119, Nueva Ecija, Philippines; nnmanluluphilrice@gmail.com (N.M.); rravelaphilrice@gmail.com (R.R.); norviemanigbas@gmail.com (N.M.); 2 Institute of Crop Science, College of Agriculture and Food Science, University of the Philippines Los Baños, Los Baños 4031, Laguna, Philippines; pcstacruz@up.edu.ph (P.S.C.); jehernandez@up.edu.ph (J.H.); 3 International Maize and Wheat Improvement Center, Gigiri P.O. Box 1041-00621, Nairobi, Kenya; tpparthiban@gmail.com; 4 Department of Crop Science, College of Agriculture, Central Luzon State University, Science City of Muñoz 3119, Nueva Ecija, Philippines; monique_corpuz@clsu.edu.ph; 5 Philippine Rice Research Institute-Negros, Murcia 6129, Negros Occidental, Philippines; adpalanog@up.edu.ph; 6 International Rice Research Institute, Los Baños 4031, Laguna, Philippines; s.kadaru@cgiar.org

**Keywords:** high-throughput phenotyping, rice, genetic gain, imaging, rice breeding, phenomics

## Abstract

The reliance on traditional or manual phenotyping creates significant operational bottlenecks in rice breeding due to its resource-intensive and time-consuming nature. This review focused on the significance of high-throughput phenotyping (HTP) as a promising technology that enables rapid, accurate, and non-destructive phenotyping of large populations. HTP has great potential to accelerate rice breeding by revolutionizing phenomics. This review examined the various applications of HTP in rice research, phenomics, and breeding. The use of HTP in rice has been substantiated through a range of cutting-edge technologies, such as drones, imaging systems, and sensor networks, that facilitate precise monitoring of key traits at various growth stages, assessment of responses to biotic and abiotic stresses, and the identification of genes or quantitative trait loci (QTLs) associated with essential characteristics. Also, this review discussed HTPs’ contributions to current rice breeding programs and documented notable challenges in scaling them. This review offers insights into optimizing HTP strategies to advance rice research, phenomics, and rice breeding.

## 1. Introduction

Rice, as an important staple crop, accounts for approximately 20% of the global dietary energy supply and serves as a primary food source for around 3.5 billion people worldwide [1]. However, global demand for rice outpaces improvements in yields, with an estimated mean genetic gain of only 0.92% [2], which is below the required gain (2%) to meet global rice demand in 2050 [3]. This low level of crop production could lead to food insecurity as the world population rapidly increases. However, in the last 50 years, extensive efforts in breeding and agronomy have tripled cereal yields [4]. Advancements in breeding, particularly the use of molecular tools, have the potential to increase genetic gain [5]. A key factor limiting genetic gain is the inability to phenotype large populations accurately and efficiently, particularly for complex traits such as yield and stress tolerance [6,7,8].

Plant phenotyping is the comprehensive assessment and measurement of key traits, including development, growth, resistance, tolerance, physiology, architecture, yield, ecology, and individual quantitative parameters used to evaluate complex traits [9]. Key agricultural traits are commonly phenotyped using conventional recording, which is often strenuous. Multi-location trials that are repeatedly conducted in different environments and across multiple seasons to capture the trait of interest are time-consuming and labor-intensive [10]. Phenotyping is a resource-intensive aspect of plant breeding that requires significant improvement [11], and the lack of phenotyping platforms that can identify rare and promising individuals from a large population remains a bottleneck in breeding programs [12,13,14]. Even with technological innovations that rapidly characterize genomes and computational methods that enable analysis of large datasets, the ability to measure phenotypic performance in the field rapidly and accurately remains a limiting factor in breeding and genetics [15]. Hence, a rapid and precise phenotypic assessment of thousands of breeding lines in the field is critical to accelerating the development of new and improved varieties. Recently, significant efforts and investments have been made to develop automated platforms to expedite plant phenotyping [10], thereby accelerating breeding programs.

Unlike traditional or manual phenotyping, which is often inefficient, destructive, and error-prone, high-throughput phenotyping (HTP) offers a promising alternative for phenotypic selection [16]. High-throughput phenotyping is a fast and, in many cases, non-destructive method for measuring a range of plant traits, including height, biomass, yield, tiller number, root system architecture, and stress responses [11]. It is rapidly growing and has revolutionized phenotyping procedures. While advances in genotyping have reduced annual expenditures, the costs associated with manual phenotyping have remained stagnant. Consequently, HTP offers substantial time and economic efficiencies for breeding programs when used effectively [17]. HTP allows scientists to identify and select the best lines for development more rapidly and cost-effectively. Through HTP, data acquisition and processing of large-scale phenotyping and plant phenomics have the potential to accelerate genetic improvements and promote the next green revolution in crop breeding [18]. This review examines the use of HTP for rice breeding. Unlike prior reviews focusing on HTP broadly across crops, this review focuses specifically on rice, organizing coverage by growth stage to provide a stage-specific guide for rice breeders.

## 2. Trends in HTP Technologies in Rice Breeding

Rice requires phenotyping throughout its growth stages to capture important traits throughout its lifespan. However, conducting continuous measurements and field observations is labor-intensive, especially at large scales [19]. The integration of HTP methods, however, facilitates significant temporal and economic efficiencies. Automatic classification of rice growth stages facilitates breeding and crop management within large-scale breeding programs. Accurate identification of the stages enables the collection of important agronomic data, such as days to 50% flowering and days to maturity, to aid breeding for early-maturing varieties. Knowledge of specific milestones in rice growth also enables accurate scheduling of crucial activities in the breeding program, such as counting tillers and harvesting at optimal maturity [20].

High-throughput phenotyping for rice uses multiple technologies. Image capture may be done using satellites or different sensors, including red–green–blue (RGB), infrared, near-infrared (NIR), and multispectral sensors [11]. These sensors may be mounted in fixed positions in both laboratory and field setups, or attached to a movable machine, such as a cart or a drone [21]. Marsujitullah et al. [22] used RGB cameras mounted on a drone to analyze rice growth stages. The researchers used the Support Vector Machine (SVM) machine learning technique to categorize rice into the following four stages: initial, vegetative, reproductive, and harvest, achieving 93.3% accuracy. The method is primarily intended for monitoring large areas. A similar method of image capture was employed by Murata et al. [23]. A multispectral sensor “Sequoia” was mounted on a drone that followed predetermined flight paths over the selected rice paddies. The Normalized Difference Vegetation Index (NDVI) was used to classify rice growth stages using a deep learning Convolutional Neural Network (CNN). Both previously mentioned studies ruled out satellite image classification due to insufficient detail and the small size of typical farms in Indonesia and Japan, respectively. Marsujitullah et al. [22] used a drone capture height of 500 m, while Murata et al. [23] used heights of 30 m, 60 m, and 100 m. Though this technology is primarily farmer-focused, it can also be used in breeding efforts to develop early-maturing and lodging-resistant rice.

High-throughput phenotyping can be used to monitor plants throughout their life cycle, as demonstrated by Theerawitaya et al. [24] for potted indica cultivars. The researchers used a PlantScreen^TM^ RGB Imaging Unit to capture images of the plants from various angles at six time points after planting. Together with manual measurements, the same software was used to analyze canopy width and height, plant-projected area. Canopy height and width and leaf area measurements from RGB imaging were highly correlated with manual measurements, with r values ranging from 0.87 to 0.99. A recent study [25] also developed a model that achieved 98.7% accuracy in classifying rice stages using camera images. The random forest (RF) machine learning model was used to calculate paddy height and canopy cover. The model also accounts for temperature, height, and the number of days after transplanting. However, this study used a fixed-position surveillance camera rather than a drone-mounted camera. This is noted as a limitation; however, distortion from the camera angle may lead to problems in predicting plant height and image segmentation [25].

**Figure 1:**
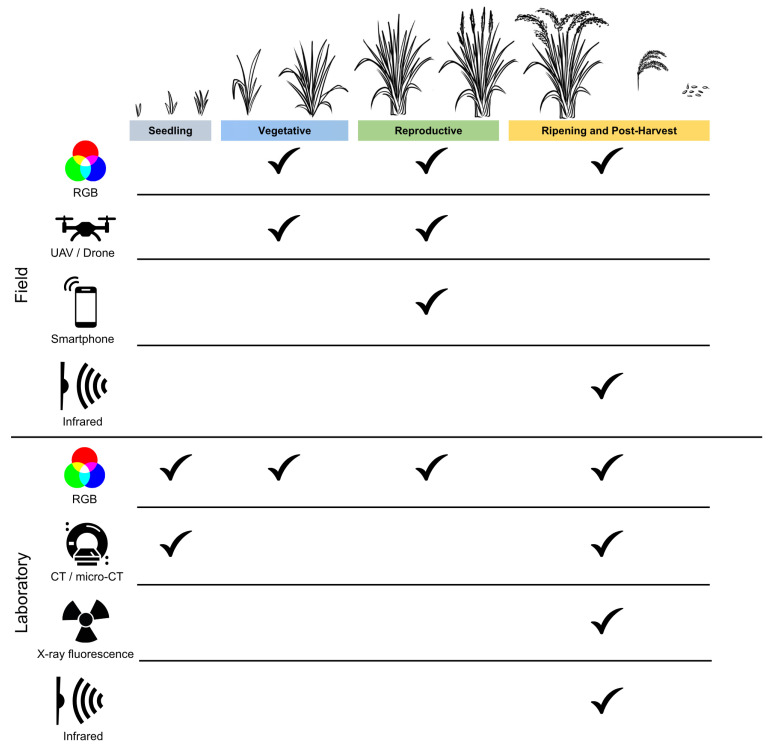
High-throughput phenotyping tool use across rice growth stages and experimental settings. Checkmarks indicate available HTP tools throughout the rice growth stage.

Scientists and breeders have developed multiple phenotyping approaches and technologies, deployed across various platforms, to efficiently collect phenotypic data on key traits and overcome the limitations of manual phenotyping. Here, we list some of the identified and applied HTP technologies used to gather data at various stages of rice plant development, as summarized in Figure 1.

### 2.1. Seedling Stage

Seedling management is a critical component of rice cultivation, particularly in the direct-seeding method, where seedlings encounter increased competition from weeds and other environmental factors, thereby underscoring the necessity of identifying seedling-related traits [26]. A primary challenge in phenotyping seedling traits is reliance on destructive approaches to measure biomass, leaf area, and other important traits. However, the development of high-throughput, non-invasive phenotyping methods that screen for traits associated with target traits, such as yield, in the early stages, has the potential to improve breeding efficiency and plant research. HTP also shortens the breeding cycle [19,27]. Since the early part of the breeding stage requires very large populations, evaluating individuals for high-yield potential before actual screening and selection can save significant time, resources, and effort through HTP. For instance, in rice, identifying and eliminating a large number of non-vigorous plants before transplanting is highly advantageous [27].

Seedling vigor in rice is associated with viability, height, stem thickness, and uniformity and is considered an indicator of improved tiller quality and yield. It could be assessed by evaluating several components, such as primary/secondary tillers, shoot length, biomass, and leaf area index [28]. The efficient selection of seedling vigor based on component traits can help advance promising lines with high-yielding potential. Seedling vigor may be assessed through HTP [27,29]. For instance, multiple genotypes were analyzed non-destructively by Anandan et al. [29] using a digital single-lens reflex (DSLR) camera, rather than traditional destructive methods that require uprooting. This enables less resource-intensive phenotyping, with the possibility of harvest recovery, compared to traditional methods that result in plant death. A high correlation was observed between the results of the two phenotyping methods, indicating that the non-destructive method could replace traditional destructive methods. This saved an estimated 80% of the time needed for phenotyping and 50% of the labor costs [29]. Targeting early seedling vigor also holds promise for breeding rice varieties with stress tolerance [26].

Other aspects of seedling growth can also be examined non-destructively with HTP. For example, root system architecture (RSA) is traditionally assessed using a destructive method by uprooting the plant. RSA is key to nutrient and water uptake, affecting crop performance. It also affects the stress response in plants, particularly under drought. However, the destructive phenotyping of RSA means that no further data can be collected from the plants. Guimarães et al. [30] also noted that HTP is not suitable for in situ root phenotyping. Despite this, HTP may be used to non-destructively phenotype roots in controlled environments. Computed tomography (CT) scans and image analysis can be used to create 3D models and to measure root traits such as length, diameter, and branching. This is limited to potted plants only in specific soils such as calcined clay. However, the recorded traits enable targeted breeding of RSA [31]. Magnetic resonance imaging (MRI) has also been used to evaluate plant roots, similar to CT scans. White neutron beam radiography and tomography may be used but degrades plant tissue, MRI and CT scans, in contrast, have no negative effect on the plant. However, CT scans are more frequently used due to their low cost and wide availability [32]. Additionally, novel HTP systems may be used to phenotype RSA of rice plants, such as in the work of Clark et al. [33]. Gellan gum was used as a growth medium to improve optical clarity, and 2D images were taken while rotating around the plant roots. From this, a 3D reconstruction of the root system architecture may be generated. However, growth in a non-soil medium may itself cause differences in RSA, and root growth in field settings may differ from the phenotype observed in the laboratory set-up. Studies may also combine manual and image-based analyses to measure a wider range of traits than a single method can [34].

### 2.2. Vegetative Stage

The vegetative stage is the first of the three phases of rice growth after seed germination. This phase begins after germination and ends at panicle initiation. Most plant growth occurs during the vegetative stage. Breeders focus on key traits during the vegetative stage, including plant height, biomass, and tiller number. The vegetative stage is a crucial indicator of the plant’s health in later stages [24].

Phenotyping of the vegetative stage can be simplified with HTP. Mano and Igawa [35] used a non-destructive phenotyping method with a time-lapse digital camera installed in the rice field, employing scale bars to measure height and diameter. The camera measurements were highly correlated with the manual measurements (R^2^ = 0.99 for plant height and R^2^ = 0.94 for stem diameter) and were accurate, though these measurements were obtained in controlled field plots rather than large-scale breeding nurseries. Field phenotyping of upland rice plant height was conducted using unmanned aerial vehicles (UAVs), commonly called drones, with a mounted RGB camera. The imaging data were used to generate a canopy height model, which was subsequently compared to manual measurements of plant height. Results indicated accurate classification of plant height, with an error of 8.839 cm. Furthermore, this was estimated to yield significant time and labor savings, with UAV phenotyping requiring 12 min of one person’s labor. Manual measurements require a full day of three-person phenotyping [36].

Similarly, Theerawitaya et al. [24] used RGB imaging to phenotype canopy width, height, biomass, and leaf area, achieving high correlations between imaging-derived measurements and manual measurements (r = 0.87–0.93 and 0.90–0.99). A positive relationship (r = 0.93–0.99) was also observed between the plant projected area in RGB images and manual leaf measurements. However, these measurements were conducted in a controlled laboratory environment. Pongpiyapaiboon et al. [19] demonstrated a digital system that generates 3D reconstructions in controlled greenhouse environments, measuring plant height, biomass, color, and leaf structure. Results showed a strong correlation between digital and manual measurements.

Tiller number and angle are important traits affecting rice grain yield, as they influence panicle number. The combination of imaging methods enabled the researchers to overcome occlusion in purely RGB-based HTP methods. Wu et al. [37] used micro-CT and RGB imaging to phenotype rice during the tillering stage and used these data to perform a genome-wide association study (GWAS) for tillering traits. The researchers also noted that the system achieved a throughput of approximately 310 plants per 24 h. A transformer-based deep learning model (TillerPET) developed by Zhou et al. [38] enables effective phenotyping of tiller number and compactness from images of post-harvest rice stubble. The TillerPET reported R^2^ values of 0.941 for tiller count and 0.978 for tiller compactness, indicating high consistency and reliability in phenotyping. On the other hand, Yamagishi et al. [39] developed a simple image-processing pipeline using UAV to estimate tillers from the number of long-growing leaves, with an error of ±3 tillers.

### 2.3. Reproductive Stage

The reproductive stage starts with panicle initiation and includes booting, heading, and anthesis. Key traits affecting grain yield become apparent at this stage. Rice grain yield is the result of a complex interplay among several factors. Spikelets, the reproductive structures on the rice panicle, determine the number of seeds per panicle, which is a key yield component that can adapt to environmental conditions. In contrast, panicle number and individual seed weight are more genetically controlled. Breeders use grain traits like total grain number, size, and weight to assess yield and quality [40]. Traits, such as high spikelet and panicle numbers, are prized for their contribution to yield.

Automated counting and imaging are often a target of HTP for rice panicles. Duan et al. [41] used multi-angle image analysis to identify and count rice panicles in a laboratory setting, despite the similarity between leaf and panicle colors. However, a recognized limitation is that rice varieties with significant visual overlap between leaves and panicles, and those with a high number of panicles, exhibit unavoidable occlusion. Similarly, Zhou et al. [42] detail the training of a model to count panicles from UAV images of rice fields, with a precision of 0.868. It was noted that this method could phenotype varieties at different stages, with significant differences in appearance.

Chen et al. [43] also used UAVs to phenotype 294 inbred rice lines, extracting panicle counts and heading dates using various machine learning models. The chosen model, YOLOv8-X, also proved capable of extracting early heading dates, with R^2^ values for the measured 10% and 30% heading dates relative to the manual measurement of 0.9387 and 0.9301, respectively.

Considerations of HTP technology availability are also present throughout the literature. Yang et al. [44] developed the PanicleNeRF v1 software for HTP of rice panicles using smartphone videos. Panicle length and volume were both estimated using the method and were strongly correlated with grain number and mass. This method enables low-cost HTP of rice panicles using readily available hardware, such as smartphones, allowing breeders and researchers with limited resources to use the software. However, noted limitations include the need to phenotype under windless conditions and a focus on individual panicles.

### 2.4. Ripening and Post-Harvest Stage

The ripening stage is the rice’s final phase of development, encompassing grain filling and maturity. Rice grains fill with starch and eventually lose moisture as the seed matures. Rice quality and yield depend on harvest timing. Ideally, harvest should occur when most seeds are mature and at full weight to maximize head rice after milling. Unripe seeds result in chalky or empty grains, whereas overripe seeds shatter easily [45].

One indicator of rice maturity is the color of the panicles and grains. Haw et al. [46] investigated the use of color analysis to predict the maturity of paddy rice for the MR219 variety in Malaysia. Images of rice florets were captured, and the hue was analyzed to determine maturity. The analysis revealed that the hue of the florets was a more reliable indicator of maturity compared to the color of the leaves. On the other hand, Wang et al. [47] used random forest regression, a machine learning technique, to measure panicle color in two rice varieties by analyzing the color of entire panicles. This method achieved high accuracy (93%) with minimal preparation, making it potentially fast and cheap. However, the method may currently be applicable only to the Jijing816 and 5Y4 varieties, as mature panicle color differs by variety.

Traditionally, breeding crops for larger kernels, a key factor in yield and consumer preference, has been constrained by the need to measure them manually. To address this bottleneck, a new method developed by Tallada and Bandonill [48] uses a flatbed scanner, software, and image analysis techniques to quickly and accurately measure kernel size. This technology is demonstrably accurate with correlation values R^2^ = 0.973–0.998. A similar technology is used by Tanabata et al. [49] to measure seed shape and size with scanner images using their developed software SmartGrain v1. This software also allows for automated counting and removal of awns and pedicels from individual grains.

Panicle structure also greatly influences yield through its effects on rice spikelet number and shape. Ikeda et al. [50] developed the PASTAR (Panicle Structure Analyzer for Rice) and PASTA Viewer software v1 for analyzing panicle structure traits, generating images of panicle branching structure to facilitate researchers’ analysis. A similar software, P-TRAP (Panicle TRAit Phenotyping) v1, was also developed to analyze post-harvest panicle structure, seed shape, and the number of spikelets [51]. The P-TRAP software is open-source, while the PASTAR v1 software requires a license. Crowell et al. [52] further claimed to improve on these panicle analysis tools in their phenotyping platform PANorama, which automatically removes awns and similar structures that interfere with panicle phenotyping. Xiong et al. [53] also developed the panicle-SEG method for panicle segmentation under field conditions, primarily for non-destructive yield estimation. Moreover, Lu et al. [54] developed a method that uses visible-light scanning and deep learning to analyze intact, unmounted panicles, eliminating the need for threshing or fixation. This method also allows for estimation of parameters that may be occluded. Su and Chen [55] further developed these methods by using X-ray CT scans to phenotype intact rice panicles. A 3D model of the panicle was then generated. This removes the need to manually mount and spread the panicle for flatbed scanning, providing more accurate in vivo rendering of panicle architecture. This method also proved effective for calculating spikelet number and seed-setting rate, with average accuracies of 100% for spikelet count and 96.65% for seed-setting rate across the eight tested samples, though validation on only eight samples limits the generalizability of this claim. However, this method requires more specialized equipment than a scanner.

Environmental changes, such as increasing temperature, directly affect grain quality. It could result in increased chalkiness, reducing rice’s market value. Chalkiness is caused by loose packing of starch granules, resulting in air pockets in the rice that appear as opaque white portions of the grain [56]. A method for analyzing grains using micro-CT scans was developed by Su and Xiao [57]. This method can detect chalkiness in both milled and unmilled rice, but it may be too costly for large datasets. Moreover, Wang et al. [56] developed an alternative method for detecting chalkiness using Epson V39 RGB photo scanners (Epson, Suwa, Japan) to capture data. The automated detection of chalkiness was performed using CNNs and Gradient-weighted Class Activation Mapping (Grad-CAM). The software was used to generate a heatmap of areas affected by chalkiness on rice grains, and Grad-CAM produced the most accurate results. However, the HTP method has certain limitations, notably the need to conduct separate training sessions for polished and unpolished rice.

HTP can also be used to reduce the costs of phenotyping non-visible traits in rice grains. Near-infrared hyperspectral imaging has also been used to differentiate rice varieties in seed form, as demonstrated by Kong et al. [58]. Meanwhile, X-ray fluorescence (XRF) enables non-destructive phenotyping of grain samples for zinc content. The machine does not require intensive training to operate and shows significant correlation (0.963–0.993) with the gold standard. It also enables rapid phenotyping, processing 250–300 samples per day, compared to other methods that process 100 samples per day [59]. This allows for effective varietal selection and breeding for high zinc content. A similar approach is used by Mohapatra et al. [60], who employed NIR reflectance spectroscopy to quantify the protein and essential amino acid content of rice.

Lodging reduces overall yield [61]. Phenotyping for lodging resistance is challenging since creating artificial wind conditions is costly. Using HTP instrumentation to assess mechanical strength and then quantify it with a mathematical relationship is critical [62] to effectively phenotype lodging resistance. In this case, Liu et al. [63] used thermal detection of lodging via infrared with the aid of UAV flights. Infrared detection is more reliable than from RGB images alone. Similarly, Wang et al. [64] developed a low-cost, non-destructive HTP method by combining UAV-based hyperspectral measurements with machine learning to estimate yield and lodging.

Several open-source or publicly available methods in this section may be used by other breeding programs [49,51,52,53,54,56,57]. Some of the promising HTP methods are panicle-SEG developed by Xiong et al. [53] for yield estimation in the field, and the methods for detecting chalkiness developed by Wang et al. [56]. Both of these methods are publicly available and have been tested in multiple varieties. Additionally, these methods use RGB imaging that is readily available and requires little specialized equipment.

## 3. Contributions of HTP in Developing Rice for Stress Environments

Environmental factors affect rice production yield [65]. Abiotic stresses, such as heat, drought, and high salinity, are exacerbated by climate change and are especially prominent in major rice-producing areas in Asia [66]. Thus, the development of stress-tolerant lines is essential, and breeding for stress tolerance requires efficient characterization of plant stress response [11]. Plant responses to stress are complex and can be positive, negative, or neutral, depending on specific traits. Consequently, phenotyping for abiotic resistance is complex and challenging [67].

Biotic stressors, such as pests and diseases, also present major challenges for rice farming, causing significant declines in yield [68]. The majority of studies involving biotic stressors make use of manually derived infection rates and scoring tools that are both time-consuming and subjective [69].

Traditional phenotyping methods are laborious, time-consuming, and prone to error. However, advances in HTP technologies have enabled rapid and precise phenotypic assessments under both controlled and field conditions. These technologies provide detailed, non-destructive measurements of critical traits, such as root and shoot architecture, chlorophyll content, and canopy temperature, which significantly improve breeding programs aimed at enhancing rice resilience to biotic and abiotic stresses [70]. Here, we discussed the different HTP technologies developed and used for each abiotic stress. These technologies were summarized in Table 1.

### 3.1. Drought Stress

Characterization and identification of drought response in rice plants are critical in breeding. Drought stress is a result of reduced available water and cellular dehydration, which can be assessed using canopy temperature and stomatal conductivity as indicators of water status [67]. Duan et al. [71] used RGB imaging to analyze rice plant response to drought as a more objective alternative to traditional visual scoring methods, which can vary across raters. Four parameters related to greenness levels and plant shape ratios reliably differentiated drought-tolerant and drought-susceptible accessions under laboratory conditions. Perimeter-area ratio and greenness-plant area ratio are also effective for field quantification of drought response. Meanwhile, Kim et al. [10] used the PlantScreen^TM^ (Photon Systems Instruments, Drásov, Czech Republic) robotic XYZ system to investigate drought stress in rice by using a combination of imaging techniques, including RGB, NIR, and infrared, to capture various morphological and physiological traits of plants under drought stress. RGB imaging showed that the indicators of drought-tolerant phenotypes include projected plant area, plant color, compactness, convex hull area, and eccentricity. NIR intensity was found to be a good indicator of drought stress, correlating with reduced plant area. Drought-susceptible plants consistently had a higher NIR intensity compared to drought-tolerant plants. Infrared imaging was also used to determine plant temperature, with drought-tolerant plants having lower temperatures on average. This approach effectively identified drought-tolerant and drought-susceptible rice genotypes, as validated with the drought-tolerant mutant *osphyb*.

Difficulties in using HTP methods, particularly infrared technology, to determine drought stress under field conditions persist. The need for consistent baseline measurements and environmental conditions makes it difficult to use infrared HTP to measure aspects such as plant temperature [81]. However, Konate et al. [73] used infrared thermography to determine drought stress in field conditions by calculating a crop water stress index from the measurements. This allowed for the identification of a drought-tolerant line with high yield. Jiang et al. [72] demonstrated the use of high-efficiency, high-frequency UAV-based image acquisition to quantify the dynamic drought response of rice populations under field conditions. Leaf rolling was used as a measurement of drought response. Compared with 1.5 h spent by three manual raters, HTP reduced phenotyping time to about 5 min of UAV flight time. The HTP-based information also enabled GWAS of drought-tolerance mechanisms. Similarly, Laraswati et al. [82] used image-based phenotyping to assess drought stress levels. Several factors were identified as strong predictors of drought stress in the two environments tested, underscoring the need to phenotype the effects of abiotic stress on rice across multiple environments.

### 3.2. Heat Stress

Increased temperatures and extreme heat events pose a significant threat to rice yields. Elevated night temperatures can increase dark reaction rates, leading to greater use of photo-assimilates that could otherwise support reproductive growth [83]. Daytime heat stress affects productivity, especially during the pre-anthesis and anthesis stages. Heat-tolerant rice must maintain its photosynthetic capacity under heat stress. Robson et al. [74] developed an HTP method to screen for photosynthetic heat tolerance by measuring chlorophyll fluorescence across different temperatures. This HTP method can scan hundreds of plants per day. This allowed researchers to model the response of photosystem II to temperature and estimate the heritability of heat-tolerance traits in both *Oryza glaberrima* and *Oryza sativa*.

### 3.3. Salt Stress

Salt stress is another abiotic factor that is usually unavoidable, depending on location. It adversely affects rice growth and yield. Developing salt-tolerant rice varieties requires an effective screening method to identify suitable genotypes [67]. Assessment of salinity tolerance in rice focuses on morphological traits, such as shoot weight and sodium ion concentration, shoot Na+/K+ ratio, plant survival rate, leaf damage and area index, and root bypass flow [84]. Assessment of plant biomass is often destructive and complicates the measurement of growth response dynamics.

Recent advancements in image-based phenotyping offer a noninvasive approach to monitor plant responses and determine salt stress [77]. For instance, Siddiqui et al. [79] used infrared thermography to phenotype rice plant responses to salt stress. Plant temperature as measured through infrared thermography is a significant sign of salt stress, with plants under salt stress showing increased temperatures. Relative water content and stomatal conductance were significantly correlated with infrared image temperature. Also, Sakinah et al. [76] used image-based phenotyping to assess rice plants’ responses to drought and salinity. A stress tolerance index (STI) with multivariate analysis was used to select the most important traits. Both morphometric and colorimetric traits could be used for selection in plant breeding. More colorimetric traits predicted drought and salinity tolerance in rice. Image-based phenotyping, combined with STI and multivariate analysis, provided a rapid, efficient method for screening rice for drought and salinity stress. Hairmansis et al. [77] used the LemnaTec 3D Scanalyzer system to phenotype IR64 and Fatmawati plants under salt stress and found that the shoot area was reduced. This approach avoids the destructive method of seedling phenotyping, specifically in measuring biomass. Al-Tamimi et al. [78] also used the same system at the same location to phenotype indica and aus rice accessions under salt stress, measuring the relative growth and transpiration rates, and transpiration-use efficiency. HTP systems may be developed for specific analysis.

### 3.4. Cold Stress

Developing cold tolerance for rice can expand the geographic range where rice may be cultivated, allowing for higher rice production [85]. Few HTP methods for cold stress tolerance are available for rice. Moura et al. [80] used RGB imaging of roots and a chlorophyll meter, both non-destructive techniques to phenotype total root length of seedlings subjected to cold stress and measure the chlorophyll content of rice leaves under cold stress, respectively. However, the majority of studies on cold tolerance in rice involve manual assessment for seedling survival [16], electrolyte leakage [86], leaf discoloration [87], and other traits.

High-throughput phenotyping for cold tolerance has been developed for non-rice crops, such as maize, using RGB imaging and hyperspectral imaging [88]. Non-specific HTP tools for seedling vigor have also been developed [27,29]. Future HTP for cold tolerance in rice could leverage these methods.

### 3.5. Biotic Stress

Biotic stresses caused by pests and diseases that reduce yields and lower grain quality are major concerns among rice farmers. HTP of biotic stress may allow for early detection [89], assessment of severity [90], or classification between healthy and diseased plants [91]. However, breeding applications require not just high overall accuracy but also low false negative rates for rare or early-stage infections, which is not always reported in the studies reviewed here.

Phadikar et al. [92] used Bayes’ and SVM classifiers to determine the extent of leaf brown spot and leaf blast infections in images of rice leaves in the field. Accuracy rates of 79.5% for the Bayes classifier and 68.1% for the SVM classifier were achieved. However, because it relies on pixel color and hue, shadows and non-disease discoloration can lead to inaccurate results. Similarly, Prajapati et al. [93] used K-means clustering and SVM to classify infected bacterial leaf blight, brown spot, and leaf smut from images of rice leaves in a controlled environment. The highest accuracy achieved was 93.33% on the training dataset, with the lowest being 73.33% on the test dataset.

Video detection of rice pests and diseases is also possible, as demonstrated by Li et al. [94]. Detection of rice sheath blight, rice stem borer symptoms, and rice brown spot in the field was conducted on RGB video data using a custom deep CNN. However, detection confidence was low, and manual calculations were still needed to determine pest and disease severity.

Zhou et al. [95] used a combination of two machine learning models, K-means clustering algorithm and Faster R-CNN, to detect rice disease from images taken in the field. The model was able to detect rice blast, bacterial blight, and sheath blight in under 1 s for all diseases, with accuracies of 96.71%, 97.53%, and 98.26%, respectively. Rahman et al. [68] developed a two-stage CNN method of identifying and classifying eight different rice diseases from images taken under both field and laboratory conditions. Using this method, 93.3% accuracy was achieved despite the model’s smaller size. Bari et al. [91] also used deep learning to create a rice disease diagnosis system, capable of detecting rice blast, brown spot, and hispa with accuracies of 98.09%, 98.85%, and 99.17%, respectively. RGB images collected by researchers and from previous datasets were used to train the model, comprising images taken both in the laboratory and under field conditions.

HTP methods for detecting specific diseases are also available. Delgado et al. [96] used UAV-mounted multispectral cameras to phenotype 4588 unique rice genotypes in the field for rice Hoja Blanca virus infections. The SVM model was found to be more sensitive, with a rate of 0.74, as compared to the breeder scores.

Zhang et al. [90] used visible-NIR hyperspectral imaging in a laboratory setting to image rice plant leaves infected with bacterial blight. Leaves were collected and imaged over 43 days of infection, and disease severity was estimated using a custom deep learning model. A rice leaf spectral index was also developed for future assessment of bacterial blight. Classification accuracy of healthy and diseased leaves reached 92.43% and R^2^ = 0.9619, while severity estimation achieved 89.13% identification rate. Bai et al. [97] used UAVs to capture multispectral and RGB images of rice in the field and trained a detection model for rice bacterial blight using both image data and temperature data. R^2^ of 0.86 was achieved with the most accurate model.

HTP for rice sheath blight was evaluated using spectroscopy by Conrad et al. [89] and Zhang et al. [98]. Conrad et al. [89] used NIR spectroscopy for early detection of rice sheath blight using the machine learning tool SVM, together with the model with the highest accuracy. This resulted in an overall testing accuracy of 86.1%. Zhang et al. [98] used hyperspectral imaging to detect regions affected by sheath blight on the rice stalk, achieving an accuracy of 98.42% at the pixel level and 95.92% at the patch level.

## 4. Opportunities of HTP for Rice Genetic Improvement

High-throughput phenotyping accelerates the collection of important phenotypic traits that manual or traditional phenotyping would otherwise require significant time and labor to obtain. This characterization has also been shown to be more efficient and cost-effective than traditional phenotyping methods, as HTP enables the assessment of more plants in less time [17,99]. This allows for further optimization of breeding operations. HTP technology enables rapid collection of phenotypic data throughout the plant life cycle, a task that would otherwise be tedious for breeders or researchers, especially under field conditions [100]. In HTP, image- or sensor-based approaches also provide non-destructive, non-invasive alternatives to traditional methods, such as biomass measurement, which requires uprooting and destroying the plant [40]. Developed HTP can also completely automate the phenotyping process, making it faster, more efficient, and replicable, removing possible human error from the equation. Phenotyping may use subjective scales or scoring methods that vary from person to person. This leads to difficulties when conducting precise studies such as GWAS. HTP can remove subjectivity from phenotyping, enabling more precise measurements [101]. Image-based phenotyping also has the potential to be more cost-effective than traditional methods in the long run due to reduced labor costs, increased scalability, and the possibility of early-stage selection, resulting in faster breeding cycles and higher success rates in identifying stress-tolerant rice varieties [17].

Rice breeding offers many opportunities for HTP’s application throughout the plant’s growth. It allows screening of large breeding populations at the early stages [100]. HTP can quickly and accurately identify varieties with desirable traits, including those that are difficult to measure with traditional methods [24]. These traits, such as plant temperature [81] or chlorophyll fluorescence levels [74], may not be immediately visible or are impossible to measure using traditional field phenotyping. HTP can also identify plants suffering from stress before it becomes visually apparent [102]. HTP can be used to evaluate previously untested genotypes from training data, using NIR spectroscopy data to predict future performance [103]. It is also useful for keeping pace with faster breeding cycles, such as rapid generation advancement, requiring more phenotyping in a shorter time [101]. This positions HTP as an important component for accelerating genetic gain.

High-throughput phenotyping can facilitate fast-tracking genetic studies of important traits in rice by addressing phenotyping bottlenecks in traditional QTL mapping. By combining HTP with high-density genetic maps, researchers can identify genomic regions controlling complex traits in rice (Table 2). HTP allows researchers to phenotype larger datasets, which are vital for analyses such as GWAS. Results from HTP may also enable the identification of candidate genes affecting a specific trait, such as salinity tolerance [78]. Despite the challenge of obtaining large-scale phenotypic data vital for rice improvement and genomic experiments [104], advanced technologies offer solutions that paved the way for more efficient and advanced methods of collecting and processing large-scale data, such as mechanized phenotyping platforms in lab and field, that have benefited genetic and genomic studies in rice [67].

HTP can be used to inform genomic selection by providing the high-throughput data needed for genomic prediction. Genome-wide markers are associated with changes in phenotype, and subsequently, breeding values can then be calculated using genome marker data. A similar principle called phenomic selection allows the prediction of phenotype from a training set at a central location, as demonstrated by Zhu et al. [103]. NIRS data from a central soybean panel, along with seed yield, thousand-seed weight, and plant height, were collected and used to train a prediction model. The model could then be used to predict seed yield, thousand-seed weight, and plant height from the NIRS data of the experimental data set and was found to be competitive with genomic prediction.

## 5. Notable Challenges of HTP and Implications in Rice Breeding

Challenges of cost, expertise, and scalability persist in HTP. The costs of acquiring HTP tools and equipment, as well as the required personnel training, remain a major concern. In breeding programs, the shift from traditional phenotyping to HTP-based methods requires a significant initial investment [17]. Furthermore, the high level of morphological plasticity in plants poses an additional challenge when using sensors for HTP and crop phenomics compared with their use in animals [99]. In resource-limited environments such as high-salinity and drought-prone settings, other notable challenges include a lack of specialized equipment, software, and personnel, which hinder data collection and processing [101].

Cost remains a primary limiting factor to the widespread use of HTP, especially in public and national breeding programs in developing countries. Breeding programs, especially in countries with low labor costs, may still find it more cost-efficient to hire workers for manual phenotyping rather than purchase specialized equipment and conduct the required training to shift to HTP. The development of software for HTP data analysis was also identified as a major cost component when shifting to HTP [133]. However, the development of automated systems may still be prohibitively expensive, with Poorter et al. [134] estimating costs of €60,000–€120,000 for systems with lower degrees of automation, €350,000–€500,000 for fully automated systems, and €3,000,000 for optimized systems. Additionally, costs are also associated with the repair and replacement of HTP equipment. Reynolds et al. [133] noted an expected UAV lifespan of 2 years and a sensor lifespan of around 4 years due to obsolescence. The development of software for HTP data analysis was also found to account for the majority of the cost of the shift to HTP. Data storage has also been cited as a cause for concern. Some HTP methods, especially multispectral imaging, generate multiple gigabytes of data per session, requiring specialized storage and processing [135]. It has also been argued that the phenotyping bottleneck has shifted from image capture to interpretation [67].

Environmental conditions can also hamper sensor use. Sensitivity to environmental factors is a disadvantage of imaging sensors that rely on visible-spectrum light, infrared, fluorescence, and depth information [136]. In these cases, continued sensor use may result in inaccurate data. However, HTP-based data in controlled or laboratory settings may not be accurate under field conditions, and HTP data extrapolated from laboratory settings may be flawed [101]. Poorter et al. [134] further mentioned difficulties in maintaining and calibrating HTP equipment when on fixed platforms.

Standardization and reproducibility of HTP data and results further complicate phenomics research. The UAV-based imaging and hyperspectral sensors often yield inconsistent results due to variations in sensor configuration, imaging conditions, and environmental factors [137]. There is a dire need for a universal data-collection protocol to facilitate cross-study comparisons and large-scale implementation [138]. The massive data generated by HTP require advanced computational tools, which can create data-processing bottlenecks [139]. Adoption of cloud computing solutions that can facilitate large-scale analysis and adoption remains limited [140]. It is important to consider emerging technologies, such as blockchain and data storage tools, for data tracking; however, their application in phenomics remains in its infancy [141].

Current HTP technologies have also been perceived as not breeder-oriented. Deery and Jones [142] criticized the field of HTP development as being driven more by available technology than by breeders’ requirements. Part of the criticism is that many HTP technologies are focused on fundamental crop physiology rather than more efficient and informative traits for breeding and crop improvement. HTP methods may also be developed for traits that are easily scored visually, raising the question of efficiency; HTP methods that evaluate non-visual traits or traits that are highly subjective may be more useful for the breeder. Barriers such as these prevent the widespread use of HTP to inform decision-making in plant breeding.

Another limiting factor is the need for computational infrastructure, robust analysis pipelines, and data analysis experts to process images and extract meaningful information to inform breeders’ decisions. Difficulty in handling large volumes of data [143] and the lack of integrated pipelines [144] were also cited as reasons for non-adoption of HTP.

RGB (visible-light) cameras are often used in HTP due to their availability, accessibility, and ease of use compared to other imaging technologies [145]. RGB data can also be combined with depth data in RGB-D [146] and micro-CT data [37]. Other imaging techniques, such as infrared (IR), NIR, XRF, and others, are more specialized and less applicable across the plant’s general growth stages. RGB sensors typically also have higher resolution and sensitivity than other sensors, while remaining on the lower end of the cost spectrum [136]. Additionally, RGB data can serve as a supplementary sensor, combined with other imaging technologies, to phenotype a broader range of traits, including those that require 3D modeling [145].

In general, many HTP systems for rice consist of imaging software combined with a machine learning method used to automate phenotyping from the data. This raises the possibility of using the same images to phenotype different traits, such as biomass and drought stress, simultaneously. This may reduce storage and the time spent on image capture.

HTP has transformed rice phenomics and plant science more broadly, yet significant challenges hinder its wider application in research and breeding. Addressing limitations in cost, scalability, standardization, and data processing is crucial to harnessing the maximum potential of HTP in rice research and breeding. Research should prioritize standardizing phenotyping protocols, improving artificial intelligence (AI) models, expanding cloud computing adoption, and adapting field-based approaches to overcome challenges [147].

## 6. Future Directions of HTP in Rice Research and Breeding

The speed and ease of use of HTP allow an objective, trait-based selection of promising rice lines. This also prevents researcher subjectivity from affecting phenotyping results and increases accuracy [17]. HTP tools provide more accurate associations between phenotype and genotype, enabling improved precision breeding techniques based on molecular markers. This is increasingly demonstrated in GWAS studies, which seek to identify candidate genes that affect desirable target traits, such as yield, grain size, and flowering time [67].

High-throughput phenotyping increases the ease of accelerated breeding by providing tools for large-scale phenotyping over a short period, thereby shortening the breeding cycle [148]. Increasing the number of breeding cycles per year accelerates selection and breeding. Additionally, HTP enables breeders to expand the scope of their breeding programs without a significant increase in cost, allowing simultaneous evaluation of more crop lines and resulting in higher selection intensity [17]. However, few to no studies have demonstrated the use of HTP in rice speed breeding specifically, though studies have used HTP in speed breeding for wheat [149].

High-throughput phenotyping also increased interest in crop modeling and simulation, which use less time and fewer resources than physical breeding programs. HTP-derived phenomic data, such as canopy temperature, leaf area index, and biomass estimates, can be used to parameterize and validate crop simulation models, improving their predictive accuracy under stress conditions. Specifically, progress has been made in rice models that account for abiotic stress. For example, ORYZA v3, a rice simulation model, was developed with increased focus on drought and nitrogen deficiency stress [150].

### 6.1. Cost and Scalability

The high upfront costs of shifting from traditional to HTP are a significant barrier to its widespread adoption. Individuals and smaller research institutions generally do not have the capital required to transition fully to HTP [135]. HTP costs also include maintenance and user training, which may not be initially considered.

In improving the scalability of HTP systems, integration of currently existing technologies, such as the use of smartphone-based sensors, should be conducted to take advantage of smartphone accessibility to researchers [151]. Additionally, developing HTP tools for multiple environments that are not hyperspecialized can improve HTP’s scalability. However, these tools entail a trade-off, requiring more manpower to operate and analyze [18].

Breeding programs with limited budgets are generally slow to adopt HTP, such as those found in developing countries. Due to the concerns about costs, it is necessary for breeders to carefully consider the relative benefits of their chosen HTP methods. Systems with lower automation and throughput are generally lower-cost, with Poorter et al. [134] citing approximate costs of €60,000 to €120,000 as compared to €3,000,000 for high-end systems. Specialized HTP facilities are generally expensive and, due to the need for more controlled conditions, are not always practical for breeders that are focused on field phenotypes. Ground vehicles equipped for HTP generally have higher costs than comparable handheld devices or UAV [133]. Additionally, ground vehicles are less portable, which is a primary concern for a breeding program with a limited budget, as it may be necessary to transport the HTP device to multiple locations. Handheld devices have reduced hardware costs of HTP, and they are portable and reusable. However, the burden then falls on the operator for the throughput, as operators still need to move through the fields [133].

Of the available HTP methods, sensors mounted on UAVs appear to be one of the more cost-effective HTP tools. UAVs enable phenotyping in field conditions and are highly portable, allowing breeders to use the same UAV to phenotype multiple locations. Commercial UAVs have cost-effective options for breeding programs and can use various sensors such as RGB, multispectral, and thermal sensors for phenotyping [152]. In wheat, around 300 plots could be phenotyped within the flight time of 15 min, producing a throughput of around 1200 plots per hour [153]. Another measurement of throughput was able to phenotype 50 to 100 hectares of wheat fields per day using RGB sensors mounted on commercial drones. Additionally, the costs of the UAV and integrated RGB cameras were generally under $2000 [154].

### 6.2. Data Analytics and Storage

High-throughput phenotyping generates large volumes of data, raising additional concerns about storage and data analytics. Partly due to the differing needs of HTP users, there are very few standardized HTP methodologies, with different image acquisition and processing software used, as demonstrated by Bongomin et al. [155]. This makes it difficult to replicate and validate HTP experiments. Another concern is the variability in environmental conditions during plant phenotyping, especially in field settings.

Consolidated databases of phenotype data have been developed, though its implementation still faces challenges. Data storage options include cloud-based databases that allow for easier collaboration, federated databases, and high-performance computing clusters [151]. Some of the challenges in database maintenance include the lack of a specific standardized methodology and data for HTP. One proposed method of handling HTP-generated data is the FAIR (findability, accessibility, interoperability, and reusability) principles proposed by Wilkinson et al. [156]. These propose that metadata and protocols should be readily available and accessible when datasets are made public. Another standard that may be used is Minimum Information About Plant Phenotyping Experiments (MIAPPEs) proposed by Krajewski et al. [157]. Several rice-specific genotype and phenotype databases have been established, including Oryza Tag Line [158], OryGenesDB [159], and MBKBase Rice [160]. However, these databases, as of the writing of this article, are unmaintained and in the case of Oryza Tag Line and OryGenesDB, potentially inaccessible.

Due to the increased volume of phenotype and genotype data generated, concerns about data handling and interpretation have also been raised. Efficient data analysis is crucial for HTP to have an impact on breeding programs. Machine learning and artificial intelligence tools have been increasingly used to link raw image data to phenotypic traits and to quantify differences among rice varieties [67].

## 7. Conclusions

High-throughput phenotyping allows rapid, accurate, and non-destructive measurement of a variety of plant traits. HTP reduces the burden of time-consuming manual measurements. HTP thus enables breeders to accelerate their breeding programs, resulting in increased rates of genetic gain. HTP is also less susceptible to human error, enabling breeders to collect accurate data from larger populations. HTP techniques have the potential to be highly accurate, with multiple studies reporting accuracy of 90% or higher, and correlation coefficients (R^2^) above 0.9 when compared to manual measurements. HTP tools are used for rice at different growth stages, traits, and stress responses. These tools range in purpose from automatic detection and measurement to machine learning methods. HTP tools were also successful in detecting major QTLs associated with important rice traits. However, challenges in implementing HTP, such as high start-up costs for tools, equipment, and personnel training, the need to train automated tools on specific datasets, harmonization with AI, and the need for robust data storage and analytics tools, should be addressed to maximize its potential in revolutionizing rice research, phenomics, and rice breeding.

UAV and handheld HTP are the more promising and versatile HTP technologies, being useful for phenotyping a broad range of traits. The integration of AI and machine learning also allows for a greater variety of downstream HTP uses, such as genomic and phenomic selection. Some of the critical gaps in the research include the lack of integration of HTP in speed breeding programs, the cost and training barriers preventing the widespread use of HTP, and the need for collaborative tools and databases for utilizing HTP data across projects. An increased focus on automation, reducing the need for specialized training for users or preparation of individual plants for HTP, and scaling HTP use for large and low-budget breeding programs are recommended as focus for future studies.

## Figures and Tables

**Table 1:** High-throughput phenotyping methods used to measure abiotic stress response.

Abiotic Stressor	Method	References
Drought	RGB image analysis using vegetative indices	[71]
	RGB image analysis using UAV detection of leaf rolling	[72]
	RGB, NIR, and infrared imaging using robotic system	[10]
	Infrared analysis using crop water stress index	[73]
Heat	Chlorophyll fluorescence measurement for photosynthetic heat tolerance	[74]
	Infrared measurement of temperature and chlorophyll fluorescence	[75]
Salt	RGB image analysis using stress tolerance indices	[76]
	RGB image analysis using Lemnatec Scanalyzer 3D	[77,78]
	Infrared thermography for leaf and plant temperature	[79]
Cold	RGB image analysis of root length and chlorophyll meter use	[80]

**Table 2:** Candidate genes and associated traits identified in rice HTP studies.

Candidate Gene	Associated Trait	HTP Method Used	References
*DST*	grain number	high-throughput rice phenotyping facility using RGB and X-ray CT imaging and automated yield trait scoring	[105,106]
*EL5*	root growth, grain protein content	hyperspectral analysis	[107,108,109]
*glx-1*	stress tolerance, grain yield, grain protein content	hyperspectral analysis	[109,110]
*GS3*	grain size, grain length, grain width	high-throughput rice phenotyping facility using RGB and X-ray CT imaging and automated yield trait scoring	[106,111,112]
*Hd1*	plant height, flowering time, yield, plant compactness	high-throughput rice phenotyping facility using RGB and X-ray CT imaging and automated yield trait scoring	[106,113]
*MADS29*	grain filling	high-throughput rice phenotyping facility using RGB and X-ray CT imaging and automated yield trait scoring	[106,114]
*NAL1*	leaf width	specialized leaf scorer using RGB line-scan camera	[115,116]
*NAL3*	leaf shape, leaf width	specialized leaf scorer using RGB line-scan camera	[116,117]
*ORR2*	cytokinin metabolism, morphology, grain protein content	hyperspectral analysis	[109,118]
Os03g16130 *	potential salinity tolerance, transpiration use efficiency	RGB imaging using LemnaTec 3D Scanalyzer	[78]
Os05g39870 *	potential salinity tolerance, transpiration use efficiency	RGB imaging using LemnaTec 3D Scanalyzer	[78]
Os05g39900 *	potential salinity tolerance, transpiration use efficiency	RGB imaging using LemnaTec 3D Scanalyzer	[78]
Os05g46320 *	potential salinity tolerance, transpiration use efficiency	RGB imaging using LemnaTec 3D Scanalyzer	[78]
Os05g47670 *	potential salinity tolerance, transpiration use efficiency	RGB imaging using LemnaTec 3D Scanalyzer	[78]
Os11g05930 *	potential salinity tolerance, transpiration use efficiency	RGB imaging using LemnaTec 3D Scanalyzer	[78]
Os11g05935 *	potential salinity tolerance, transpiration use efficiency	RGB imaging using LemnaTec 3D Scanalyzer	[78]
Os11g07230 *	potential salinity tolerance, transpiration use efficiency	RGB imaging using LemnaTec 3D Scanalyzer	[78]
Os11g07240 *	potential salinity tolerance, transpiration use efficiency	RGB imaging using LemnaTec 3D Scanalyzer	[78]
*OsbZIP12*	drought tolerance, flowering time	RGB imaging using UAV and deep learning	[72,119]
*OsEREBP2*	salinity tolerance	RGB imaging using UAV and deep learning	[72,120]
*OsGH3-2*	plant height, auxin and abscisic acid regulation, drought tolerance, cold tolerance	high-throughput rice phenotyping facility using RGB and X-ray CT imaging and automated yield trait scoring	[106,121]
*OsH43*	leaf development, leaf color	specialized leaf scorer using RGB line-scan camera	[116,122]
*OsHB2*	leaf development, leaf color	specialized leaf scorer using RGB line-scan camera	[116]
*Oshox1*	leaf shape, leaf size, leaf color	specialized leaf scorer using RGB line-scan camera	[116]
*OsIAA3*	leaf length	specialized leaf scorer using RGB line-scan camera	[116,123]
*OsITPK2*	drought tolerance, salinity tolerance	RGB imaging using UAV and deep learning	[72,124]
*OsNRT1*	leaf color, nitrogen use	specialized leaf scorer using RGB line-scan camera	[116,125]
*OsPPKL3*	grain length, grain size	high-throughput rice phenotyping facility using RGB and X-ray CT imaging and automated yield trait scoring	[106,126]
*OsRCI2-5*	drought tolerance	RGB imaging using UAV and deep learning	[72,127]
*OsSRO1c*	drought tolerance, cold tolerance, oxidative stress tolerance	RGB imaging using UAV and deep learning	[72,128]
*qSW5*	grain width	high-throughput rice phenotyping facility using RGB and X-ray CT imaging and automated yield trait scoring	[106,129]
*SAS3*	flowering time, grain protein content	hyperspectral analysis	[109,130]
*SD1*	plant height	high-throughput rice phenotyping facility using RGB and X-ray CT imaging and automated yield trait scoring	[106]
*TAC1*	tiller angle	micro-CT-RGB imaging	[37]
*TH1*	lemma and palea development, grain shape, grain weight	high-throughput rice phenotyping facility using RGB and X-ray CT imaging and automated yield trait scoring	[106,131]
*YGL1*	chlorophyll, leaf color	specialized leaf scorer using RGB line-scan camera	[116,132]

* Genes identified using HTP that were not previously discovered using manual phenotyping.

## Data Availability

No new data were created or analyzed in this study. Data sharing is not applicable to this article.

## Abbreviations

The following abbreviations are used in this manuscript:

HTP	high-throughput phenotyping
QTL	quantitative trait locus
RGB	red–green–blue
SVM	support machine vector
NDVI	normalized difference vegetation index
CNN	convolutional neural network
RF	random forest
DSLR	digital single-lens reflex
RSA	root system architecture
CT	computed tomography
MRI	magnetic resonance imaging
UAV	unmanned aerial vehicle
GWAS	genome-wide association study
Grad-CAM	gradient-weighted class activation mapping
XRF	X-ray fluorescence
NIR	near-infrared
STI	stress tolerance index
AI	artificial intelligence
